# Comparative Assessment of the Efficacy of Newly Designed Multiple Variability Loop and Opus Loop for Anterior en Masse Retraction in Orthodontics: A Finite Element Study

**DOI:** 10.7759/cureus.44817

**Published:** 2023-09-07

**Authors:** Vikrant V Jadhav, Meenakshi Tiwari, Ranjit Kamble, Sunita Shrivastav, Abhishek Thote

**Affiliations:** 1 Orthodontics and Dentofacial Orthopaedics, Sharad Pawar Dental College, Datta Meghe Insitute of Higher Education and Research, Wardha, IND; 2 Rajesh Ramdasji Kambe (RRK) Dental College and Hospital, Orthodontics and Dentofacial Orthopaedics, Akola, IND; 3 Orthodontics and Dentofacial Orthopaedics, Sharad Pawar Dental College, Datta Meghe Insitute of Higher Education and Research, Nagpur, IND; 4 Mechanical Engineering, Maharashtra Institute of Technology World Peace University (MIT-WPU), Pune, IND

**Keywords:** titanium molybdenum alloy, stainless steel, finite element method, frictionless mechanics, loops

## Abstract

Introduction

Two different mechanisms are used to close the space after extractions, i.e., friction/sliding mechanics and frictionless/loop mechanics. The focus of this study is on space closure using frictionless or loop mechanics, which utilize loops to achieve the desired tooth movement. Loops are designed to increase the resiliency of the archwire so that the ideal moment-to-force ratio (M/F) of 8:1 to 10:1 for bodily tooth movement can be obtained. By incorporating various loop designs, the resiliency of archwires may be increased, and an ideal M/F ratio can be obtained.

Method

Different pre-activation bends were incorporated in the titanium molybdenum alloy (TMA) wire at alpha (canine side) and beta (premolar side), respectively. A total of 36 finite element method (FEM) models were prepared with and without pre-activation bends to evaluate the moment-to-force ratio.

Result

The results obtained from the finite element method (FEM) analysis provided valuable insights into the force and moment generated during activation while retracting the tooth. Specifically, the alpha bend at 15 degrees and the beta bend at 20 degrees produced the most desirable results for generating moments on the anterior and posterior teeth, respectively.

Conclusion

To ensure the proper utilization of the multiple variability (MV) loop, we recommend its preparation using a 0.019 x 0.025-inch TMA wire. This selection will maximize the loop's capabilities and enhance its performance in achieving the desired tooth movement.

## Introduction

Space closure is a critical aspect of orthodontic treatment to correct malocclusions. Malocclusions can be treated by either utilizing the existing space within the dental arch or by creating additional space, depending on the severity of the malocclusion. One common method to create space is by performing the extraction of the first or second premolars. The space created by extractions can be used to address various orthodontic issues, such as proclination (forward tilting of teeth), crowding (lack of space for teeth within the arch), the deep Spee curve (excessive curvature of the arch in the vertical dimension), anterior rotation correction (correcting the forward or backward rotation of the anterior teeth), and inter-arch malocclusions (problems that involve both the upper and lower arches) [1]. Two different mechanisms are used to close the space after extraction: friction/sliding mechanics and frictionless/loop mechanics. The focus of this study is on space closure using frictionless or loop mechanics, which utilize loops to achieve the desired tooth movement. A loop used for space closure needs to meet specific requirements to be effective. It should have a low force-to-deflection rate, meaning that it applies a relatively small force to produce the desired tooth movement. Additionally, the loop should have a high moment-to-force (M/F) ratio, ideally close to 10:1. The M/F ratio is a measure of the efficiency of the loop in generating the desired rotational movement of the tooth.

The position, design, and pre-action bends of the loop play a crucial role in determining the M/F ratio. Adjusting the loop's vertical length, horizontal breadth, and number of pre-activation bends can alter this ratio, resulting in improved root control and more efficient physical movement and translation of the tooth. The pre-activation bends are strategically incorporated into the loop design to achieve an adequate M/F ratio and to prevent the roots from being forced into the extraction space, a phenomenon referred to as "root dumping" [2]. During the space closure process, retraction forces can cause tipping movements of the tooth. To counteract this tipping, the anterior and posterior arms of the loop induce both anterior and posterior movement of the tooth, helping to maintain a stable and controlled orthodontic correction.

Overall, space closure using frictionless or loop mechanics is an important technique in orthodontic treatment. The design and configuration of the loops used play a significant role in achieving successful and stable tooth movement while maintaining the health and integrity of the surrounding structures. The impact of the force system on the tooth and the subsequent responses of the surrounding structures characterize orthodontic movement. To release physiologically advantageous forces that do not continuously alter the stress areas of the periodontal ligament, the forces must be continuous and the center of rotation of the tooth must remain constant. Because of this, it is difficult to close off space effectively, yet it is possible to do so when the force system is under control and predictable. The successful achievement of orthodontic movement depends on understanding the impact of force systems on the tooth and the subsequent responses of the surrounding structures. Orthodontic movement involves applying controlled forces to teeth to achieve desired positional changes. For this process to be effective and promote healthy tooth movement, certain principles must be considered [3].

First, the forces applied to the tooth should be physiologically advantageous. Physiologic forces are gentle and continuous, allowing for gradual tooth movement without causing excessive stress on the periodontal ligament, the tissue that connects the tooth to the surrounding bone. When forces are applied gradually, the periodontal ligament can adapt and remodel, allowing the tooth to move in a controlled manner. Moreover, it is crucial to ensure that the center of rotation of the tooth remains constant during orthodontic movement. The center of rotation refers to the point around which the tooth moves during its repositioning. When the center of rotation is stable, the tooth can be predictably and precisely guided to its desired position. One of the challenges in orthodontics is effectively closing off the spaces between teeth. Creating space can be necessary to correct crowding or achieve proper alignment. However, closing the space becomes challenging because applying forces to move the tooth can disturb the surrounding periodontal structures. Therefore, it is essential to carefully control the force system to prevent unwanted side effects. When the force system is adequately controlled and predictable, orthodontists can effectively close off spaces. Utilizing frictionless or loop mechanics, as mentioned earlier, is one approach to achieving this goal. By designing loops with specific characteristics, such as low force-to-deflection rates and high M/F ratios, orthodontists can apply forces in a controlled manner, ensuring that the tooth moves efficiently and with minimal disruption to the periodontal ligament [4].

The field of orthodontics has seen significant advancements in methods for computing the M/F ratio, which is crucial for understanding the efficiency and effectiveness of orthodontic treatment. Some of the methods used for this purpose include typhodonts, dynamic evaluation systems, laser holography, and finite element analysis (FEA). The finite element method is a powerful computational technique widely used in various engineering and scientific disciplines, including dentistry and orthodontics. Clough first introduced FEM in 1960. It involves creating a three-dimensional model of the tooth and surrounding structures and analyzing the complex forces and stresses that occur during orthodontic treatment. It has proven to be accurate and fast in predicting the behavior of intricate structures [5]. In dentistry, FEM was adapted by Weinstein in 1976 to assess the stresses acting on the periodontal ligament during orthodontic movement. By using software programs like ANSYS (ANSYS Inc., Canonsburg, PA, USA), MSC PATRAN-NASTRAN (MSC Software, Newport Beach, CA, USA), ALGOR (ALGOR Inc., Pittsburgh, PA, USA), and others, FEA can perform sophisticated simulations and calculations to provide valuable insights into the forces and movements involved in orthodontic treatment [5]. The application of FEM in orthodontics has significantly enhanced our understanding of the biomechanics of tooth movement and contributed to the development of more efficient and precise treatment approaches. It allows orthodontists to optimize treatment plans, design customized appliances, and predict treatment outcomes more accurately, leading to better patient care and outcomes [5]. So, this study evaluates the biomechanical characteristics and efficacy of the newly designed multiple variability (MV) loop for en-masse anterior retraction and compares it to the Opus loop.

## Materials and methods

The study was carried out at the Department of Orthodontics and Dentofacial Orthopedics, Sharad Pawar Dental College, Datta Meghe Institute of Higher Education and Research in Wardha, Maharashtra, India. The study received approval from the Institutional Ethical Committee (approval no. DMIMS(DU)/IEC/2021/578). In this study, the researchers focused on a specific type of orthodontic appliance known as loops (specifically Opus and MV). These loops were fabricated using titanium-molybdenum alloy (TMA) wires with dimensions of 0.019 × 0.025 inches. The appliance was designed to fit into a 0.022-inch slot dimension, which is a common specification in orthodontic brackets. The TMA wires used for the loops were subjected to different pre-activation bends at two locations: alpha (canine side) and beta (premolar side). These pre-activation bends are likely strategic curves introduced to create specific forces or movements when the appliance is placed in the patient's mouth. A total of 36 FEM models were generated for the study. These models were designed to evaluate the relationship between movement (likely tooth movement or adjustments) and the force applied by the orthodontic appliance.

Opus loop

Designed by Raymond E. Saitkowski, the Opus loop delivers an M/F ratio within the range of 8:1 and 9:1. The Opus loop is used in both segments and continuous archwire with no residual bends in the wire. It is observed to have the desired mechanical properties. No preactivation bends are placed in this loop [6]. The Opus loop is characterized by ascending legs set at an angle of 70 degrees to the plane of brackets. Additionally, it consists of apical horizontal legs with a length of 10 mm. The primary function of the Opus loop is to facilitate the closure of extraction spaces and aid in retraction during orthodontic treatment. It has anterior and posterior movements in opposite directions, reducing the tendency to change the occlusal plane. This design is characterized by its complex configuration, involving multiple bends and loops, which can complicate the fabrication process and lead to difficulties in placing the loop precisely. This complexity can also contribute to increased friction and binding within the bracket, potentially leading to reduced effectiveness of tooth movement and discomfort for the patient. Furthermore, the intricate nature of the Opus loop may make it more challenging to adjust during treatment, requiring additional chairside time and expertise, thus potentially increasing the overall treatment duration [6]. 

The MV loop 

The MV loop, developed by Dr. Meenakshi Tiwari and Dr. Vikrant Jadhav of the Department of Orthodontics and Dentofacial Orthopedics at Sharad Pawar Dental College, is a type of open loop similar in design to the T loop. It has specific dimensions, with an occluso-gingival height of 7 mm and a mesio-distal length of 8 mm. The MV Loop has proven to be effective for achieving en-masse retraction of the maxillary and mandibular six anterior teeth, as well as single tooth retraction, while providing optimum force. The loop design is patented in Australia (2021103077) and India (202021008633). To verify the force system and determine the appropriate degree of bends (α and β) required for bodily movement, the researchers conducted a three-dimensional FEA using specialized software. Finite element analysis is a powerful computational technique used to analyze complex structures and their responses to external forces. The MV loop has less vestibular height, making it easier for it to adapt in cases where there is a shallow vestibule. It has both horizontal and vertical arms of almost 8 mm, which allows it to control the tooth movement in both horizontal and vertical dimensions. Easier design allows fabrication of the MV loop within 3 minutes. The MV loop has a higher range of activation with less chance of breakage due to its simple design. The overall treatment is reduced with the MV loop in extraction cases. Note that the MV loop has fewer clinical trials.

Inclusion and exclusion criteria 

The inclusion criteria were as follows: models with various preactivation bends and loops designed for specific materials and moments made of TMA and stainless steel (SS); a 19 x 25-inch wire dimension with a 0.022 slot; use of FEM and data access layer (DAL) software. Materials other than TMA and SS, a 0.018 slot, and the use of other software were excluded.

Working the FEM model 

The initial step involves creating a 3D model of the wire with dimensions of 0.019 x 0.025 inches. This was done using computer-aided engineering (CAE) software SOLIDWORKS (SOLIDWORKS Corp., Dassault Systèmes, Waltman, MA, USA) or computer-aided three-dimensional interactive application (CATIA) V5R19 version 3.7 (FEBio, Salt Lake City, Utah, USA). The resulting design is then saved in stereolithography (STL) format, commonly used for 3D printing and computer-aided design (CAD). Subsequently, the STL file is imported into HyperMesh software (Altair Engineering Inc., Troy, MI, USA), facilitating meshing, where the intricate 3D structure is divided into smaller elements like triangles or quadrilaterals for surfaces and tetrahedrons or hexahedrons for volumes. This breakdown is crucial for numerical analysis. Following meshing, the model is integrated into Ansys R18.1 (Ansys, Inc., Canonsburg, PA, USA) or a similar FEA software. At this stage, material properties such as Young's modulus, Poisson's ratio, and density are defined, influencing how the material reacts to stress and strain. Geometric properties like element length and area are also specified. The characterization of the loop involves the utilization of nodes and beam elements, with beam elements being suitable for representing structures subjected to axial loading, such as wires or rods. Nodes denote the connection points between these elements. Boundary conditions are set to replicate real-world scenarios. Constraints are applied to simulate the structural fixations or restraints, impacting the movement of the model. This is followed by the application of loading conditions that encompass forces, pressures, thermal effects, and more. The interest in analyzing the loop both with and without 'alpha preactivation bends' suggests an investigation into the effects of bending before actual loading is applied. Once all boundary conditions and loading scenarios are established, the analysis is executed within the FEA software. The software employs numerical methods to compute stress, strain, deformation, and other pertinent properties of the wire loop given the specified conditions (Table 1).

**Table 1:** Different pre-activation bends in 0.019×0.025-inch TMA wire in various loop designs MV: Multiple variability loop, TMA: Titanium molybdenum alloy

MV loop	Opus loop
0^0^ α and 0^0^β	0^0^ α and 0^0^β
5^0^α and 10^0β^	5^0^α and 10^0β^
10^0^α and 15^0^β	10^0^α and 15^0^β
15^0^α and 20^0^β	15^0^α and 20^0^β

Statistical methods

This two-sided test was analyzed using the chi-square test and Student’s unpaired t-test. Both SPSS Statistics version 24.0 (IBM Corp., Armonk, NY, USA) and GraphPad Prism version 7.0 (GraphPad Software, San Diego, CA, USA) were used for data analysis.

## Results

The researchers conducted a comprehensive study involving 36 FEM models to evaluate the M/F ratio and the maximum force generated by different loop models after activation. The FEM analysis was carried out on various loop designs, considering different pre-activation alpha and beta angles in TMA wire with a dimension of 0.019 × 0.025 inches. The results obtained from the FEM analysis provided valuable insights into the force and moment generated during activation while retracting the tooth. Specifically, the alpha bend at 15 degrees and the beta bend at 20 degrees produced the most desirable results for generating moments on the anterior and posterior teeth, respectively. These specific angles were found to be optimal for achieving the desired tooth movements efficiently and effectively.

During the closure of spaces in orthodontic treatment, controlling tooth movement in all three planes (vertical, sagittal, and transverse) is of utmost importance to achieve the desired tooth positions. The tooth movements required to close spaces include vertical forces such as intrusion (to move the tooth inward) and extrusion (to move the tooth outward), rotational movements, torque (to control the root positions), and anchorage control (to prevent undesired tooth movements). While there are various methods described to control posterior tooth movement, such as tip back bends, headgear, intermaxillary elastics, and tip edge mechanics, the studied loop design offers an added advantage. It provides distal force on molars, which helps augment anchorage. Anchorage control is vital in orthodontic treatment to prevent unwanted movement of other teeth while achieving the desired space closure. The findings indicate that the 15 α and 20 β loop designs, in combination with the TMA wire, can effectively achieve the desired tooth movements and provide improved anchorage control during space closure. This loop design may offer advantages over other techniques in certain clinical situations, making it a valuable addition to the orthodontist's armamentarium for achieving optimal treatment outcomes.

**Figure 1:**
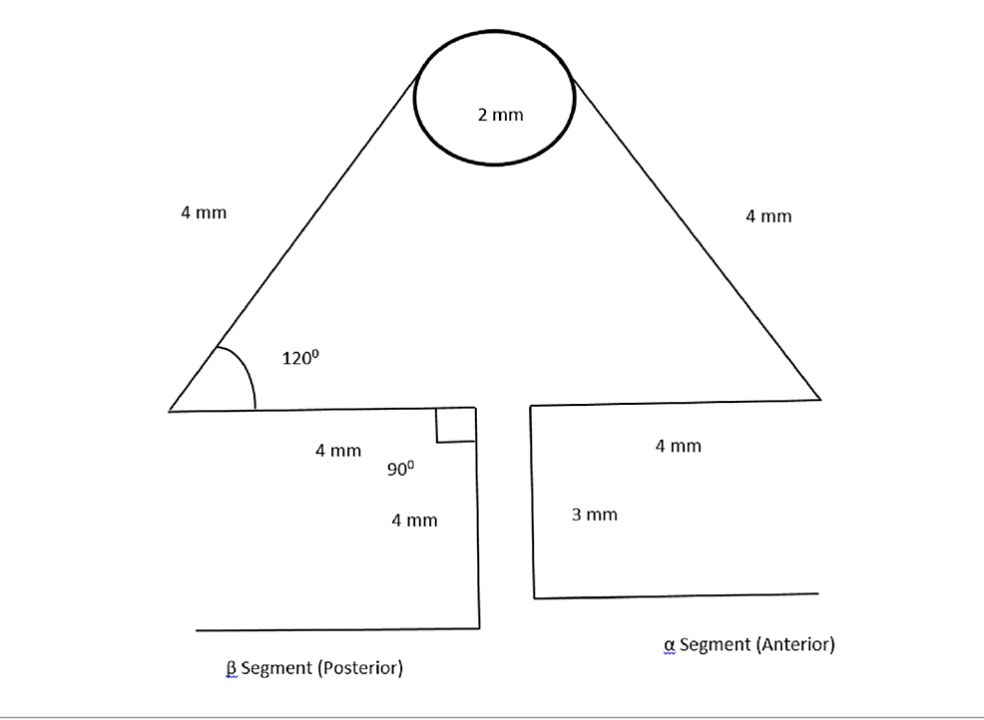
The MV loop MV: Multiple variability

The loop height is 7 mm and the width is 9 mm, with a 2 mm diameter helix. The wire cross-section used was 0.017 x 0.025 inches TMA. The 3D images were designed in a symmetrical form (Figure 1). In DAL software, the M/F ratio was calculated for the MV loop. The MV loop showed x-axis coordinates as 39.17 and y-axis as 27.31, with moment generated as 392.62 and M/F ratio generated as 8.2. This shows an adequate M/F ratio was achieved with the MV loop for translation and bodily movement. The DAL software denotes that the MV loop achieved the desired M/F ratio. With changes in the amount of force and pre-activation bends with both coordinates, the ideal M/F ratio is achieved.

**Figure 2:**
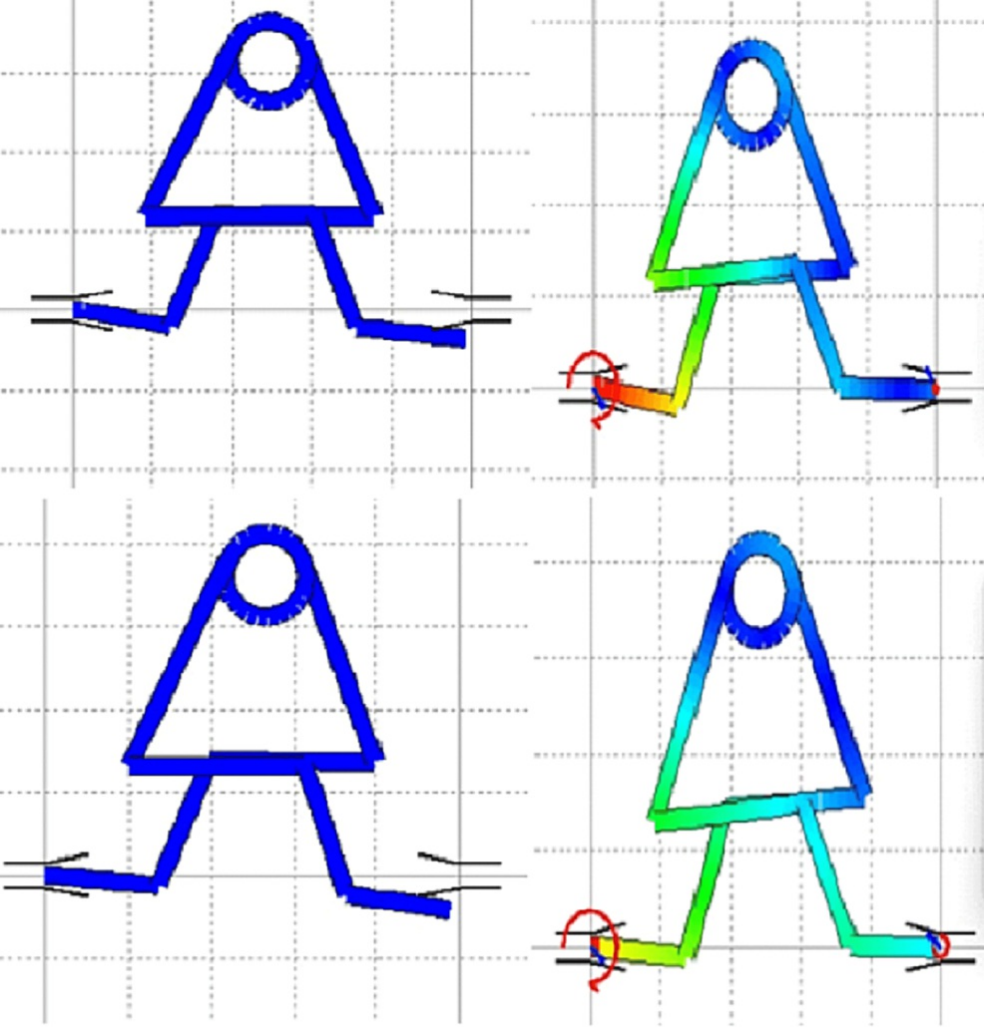
Analysis of the MV loop Analysis using the DAL software performed by authors Tiwari and Jadhav. Image generated by authors MV: Multiple variability, DAL: Data access layer

In the DAL software, the M/F ratio was calculated for the MV loop. The MV loop showed x-axis coordinates as 39.17 and y-axis as 27.31, with moment generated as 392.62 and M/F ratio generated as 8.2. This shows an adequate M/F ratio is achieved with the MV loop for translation and bodily movement (Figure 2). The DAL software denotes that the MV loop achieved the desired M/F ratio. With changes in the amount of force and preactivation bends with both the coordinates, the ideal M/F ratio is achieved.

The study involved evaluating the stress generated by the MV loop using different wire dimensions and materials (TMA and SS) through FEA. The wire dimensions tested were 0.019 × 0.025 inch and 0.017 × 0.025 inch. The FEA analysis showed that the highest amount of stress was generated when using SS wire, while the least amount of stress was observed with TMA wire.

**Figure 3:**
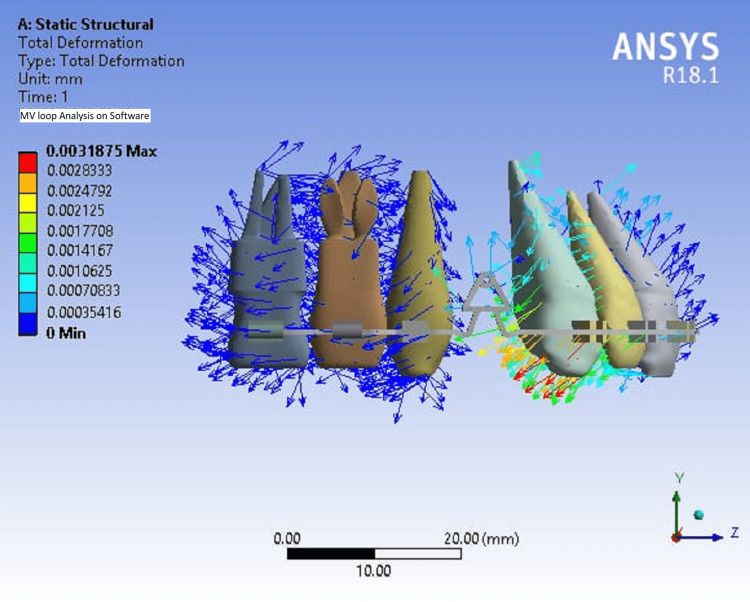
FEM analysis of the MV loop The FEM analysis was performed by author Thote. Image generated by the author. FEM: Finite element method

To further assess the stress distribution and the M/F ratio of the MV loop, physical loops were fabricated and tested under static conditions with an equivalent stress (von Mises) set at 1 MPa unit. Two different pre-activation lengths of 1 mm and 2 mm were tested. For the MV loop with 0.019 x 0.025 inch TMA wire, the observed values were 101.51 for 1 mm pre-activation and 101.56 for 2 mm pre-activation. This indicated that there was adequate stress distribution and a consistent M/F ratio of 8:1, making it a suitable choice for orthodontic use. However, when a 0.019 × 0.025 inch SS wire was used for the MV loop, the observed values were 108.52 for 1 mm pre-activation and 109.40 for 2 mm pre-activation. This indicated that there was no adequate stress distribution with the SS wire, leading to higher stress levels and an M/F ratio that may not be suitable for clinical use. The high force generated by the SS wire could potentially cause unwanted tooth movement or even fracture the loop during fabrication and insertion (Figure 3).

Based on these findings, the study recommends using TMA wire for the MV loop, as it showed minimum stress values and demonstrated that the loop would not fracture under any circumstances, ensuring its durability and effectiveness in orthodontic treatment. The MV loop with TMA wire is deemed suitable for achieving the desired tooth movements with minimal risk of complications. On the other hand, using a 0.019 x 0.025 inch SS wire for the MV loop is not recommended due to its higher force generation, which may lead to undesirable outcomes in orthodontic treatment.

The M/F ratio of the MV loop at 15-degree alpha and 20-degree beta bends is maximum and near 10 comparatively. This represents that the MV loop at 15-degree alpha and 20-degree beta pre-activation will generate the M/F ratio, which will cause translatory movement and bodily movement of the teeth subjected to force (Table 2).

**Table 2:** The MV loop M/F ratio MV: Multiple variability, M/F: Moment-to-force, TMA: Titanium molybdenum alloy

0.019 × 0.025 TMA (pre-activation bends)	Moment (mmN)	Force (N)	M/F ratio
0^0^ α and 0^0^β	11.83	1.42	7.30
5^0^α and 10^0β^	12.93	1.58	7.50
10^0^α and 15^0^β	13.75	1.60	8.00
15^0^α and 20^0^β	14.95	1.63	9.12

The Opus loop was designed to deliver inherent M/F ratios sufficient for en masse space closure via translation for teeth of average dimensions and absent marginal bone loss. Because the loop's inherent M/F is high enough, no activation bends or bends in the formed loop need to be added before insertion. The M/F is 10:1, which is adequate for bodily types of movement (Table 3).

**Table 3:** Opus loop M/F ratio MV: Multiple variability, M/F: Moment-to-force, TMA: Titanium molybdenum alloy

0.019×0.025 TMA (pre-activation bends)	Moment (mmN)	Force (N)	M/F Ratio
0^0^ α and 0^0^β	7.85	0.27	6.84
5^0^α and 10^0β^	6.32	0.20	6.61
10^0^α and 15^0^β	7.75	1.60	6.00
15^0^α and 20^0^β	6.95	1.63	6.12

## Discussion

Ricketts [7], based on a study by Lee [8], determined that 100 grams of force per centimeter of the root surface is considered sufficient for bio-progressive treatment. Bio-progressive treatment refers to a concept in orthodontics that focuses on promoting natural growth and development during orthodontic interventions [9]. On the other hand, Güray and Orhan demonstrated that for successful retraction of maxillary (upper jaw) anterior teeth, an average of 130 grams of force is optimal. Retraction is a process in which the anterior teeth are moved backward to create space or align them properly within the dental arch [10].

When intending to manipulate a tooth in a predetermined manner, it's imperative that the force exerted possess the appropriate magnitude and be applied precisely at the designated location on the tooth. Any alterations to the force's direction, magnitude, or point of application can significantly influence the nature of tooth movement, potentially leading to outcomes that deviate from expectations. The concept of optimal force in orthodontics is characterized by the force that can achieve the desired tooth movement while minimizing damage to surrounding tissues. This optimal orthodontic force proves advantageous as it enables the attainment of the intended tooth movement with minimal trauma inflicted upon the periodontal tissue. The determination of the forces and moments needed to attain these optimal force levels is essential to orchestrating successful orthodontic treatments [11].

Movement refers to the application of force to the teeth to induce their controlled displacement. The movement is described by the amount of force applied and the distance between the point of force application and the center of resistance of the tooth or any other object involved in the orthodontic treatment. The center of resistance is a point within the tooth that resists movement when force is applied [12,13]. It's important for orthodontists to carefully consider the amount of force applied during dental treatment to achieve the desired movement while avoiding any potential damage to the teeth or surrounding structures [11].

For achieving controlled bodily tooth movement, it's optimal for the M/F ratio to fall within the range of 8 to 10. This range is particularly desirable when aiming to effect the closure of extraction spaces. During the process of space closure, there is a generation of friction at the interface between the orthodontic bracket and the archwire. Friction refers to the force that acts against the motion of an object as it moves tangentially in relation to another surface. When two contacting surfaces slide against each other, various forces come into play. Friction's intensity is directly linked to the normal force exerted between the surfaces and can be mathematically described as F = μN, where μ signifies the coefficient of friction and N represents the force in Newtons. Notably, friction can be notably reduced when the archwire is optimally integrated with a loop component. This integration ensures that the wire segment moves in conjunction with the teeth, preventing relative movement between the teeth and the wire. This mechanism is particularly effective in mitigating friction and facilitating smoother tooth repositioning [11].

The FEM is a prominent analytical technique that has gained significant traction in recent times. Initially conceived for the aviation sector, the FEM has become a highly utilized tool for investigating various design aspects. This method was originally formulated to scrutinize stress distributions within intricate airframe structures. However, its applicability has extended far beyond aviation and is now extensively employed in medical and dental research. In the realm of orthodontics, FEM plays a crucial role in conducting structural stress analyses. By utilizing FEM, it becomes possible to comprehensively assess the forces generated by wire geometries within three-dimensional spaces. Over the past three decades, FEM has secured a firm footing in the domains of medical and dental research. Numerous studies have effectively demonstrated the utility of FEM in evaluating the mechanical properties of wires and their implications for orthodontic treatments. This approach has paved the way for valuable insights and a deeper understanding of the intricate interactions between wires, teeth, and the surrounding structures.

A study conducted by Pate et al. investigated forces generated in the lateral incisor area using a 0.019 x 0.025 TMA wire with 2 mm activation. The forces recorded were 1.14 N, and the M/F ratio was within the range of 6.84. Notably, no pre-activation bends were utilized in their assessment. In contrast, our study employed a 0.019 x 0.025 TMA wire with 2 mm activation, resulting in force values of 0.9058 N and an M/F ratio of 6.2. The divergence observed in our findings could be attributed to the distinct region of evaluation, specifically the canine area in our case, compared to the lateral incisor region in Patel et al.'s research [14]. Additionally, another study conducted by Arathi et al. explored similar parameters using a 0.017 x 0.025 TMA wire at 2 mm activation. Their findings indicated force values of 0.9581 N and M/F values of 3.9813. Notably, their M/F ratio was notably lower compared to our study's results [15]. These variations among different studies could be attributed to multiple factors, including the specific tooth regions evaluated, differences in wire activation, variations in wire properties, experimental setups, and potential variations in measurement techniques. Such disparities highlight the complexity of orthodontic mechanics and the need for careful consideration when interpreting and comparing findings across different studies.

The FEM study revealed that for the desired M/F ratio, the MV Loop demonstrated bodily movement for en masse retraction when the α bend and β bend were appropriately applied. The loop offered several advantages, including minimal wire bending, reduced risk of breakage, an M/F ratio of 8:1, ease of placement in shallow vestibular depth, and minimum α and β bend requirements. The biomechanical properties of the MV loop were further compared to those of the Opus loop using a 3D model. Presumably, this comparison was to assess the efficacy and superiority of the MV Loop in achieving the desired tooth movements while ensuring optimal force delivery and patient comfort during orthodontic treatment. The loop's unique design and biomechanical advantages, as demonstrated through FEA, make it a promising option for orthodontists seeking optimal outcomes for their patients.

For achieving en masse anterior retraction, it is recommended to apply a force ranging from 150 to 300 g for anterior retraction. A model using an MV loop, constructed from TMA wire with dimensions of 0.017 x 0.025 and activated with 15α and 20β angles, exhibited a maximum displacement of 9.12 units. In contrast, the Opus loop resulted in an M/F ratio of 6.12. When the MV loop was created with the same TMA wire dimensions but activated with 0α and 0β angles, it showed a minimal displacement of 7.30 units, whereas the Opus loop achieved a maximum M/F ratio of 6.84. This indicates that the MV loop requires pre-activation to achieve the desired M/F ratio, whereas the Opus loop does not necessitate pre-activation (as seen in Table 2). The TMA wires maintained more consistent moments across the deflection range. Consequently, it is evident that the MV loop inherently produces the optimal M/F ratio needed for controlled movement of the dentition. The increased height of the Opus loop relative to the MV loop contributes to elevated rates of fracture, potential tissue interference, reduced patient compliance, and heightened stress concentration. The Opus loop exhibited lower rates of force to displacement and generated a lower maximum force compared to the MV loops (as seen in Table 3).

This study aims to assess the biomechanical characteristics of Opus and MV loops in conjunction with different wire dimensions for orthodontic treatment purposes. By investigating these loop types and wire variations, the research intends to offer fresh insights into their optimal utilization to attain the highest biomechanical benefits in extraction-based orthodontic cases. Additionally, by considering combinations of wire sizes and loop pre-activations with Opus and MV loops, the study aims to provide comprehensive data that can contribute to effective and informed treatment approaches for such cases. The MV loop holds significant promise for future research endeavors. Its exceptional qualities align well with the criteria for an ideal loop design. To fully explore its potential in intricate orthodontic cases, it is advisable to strategize clinical trials incorporating the MV loop. This step could unveil a deeper understanding of the loop's capabilities and applications.

Limitations

There are several limitations to the study, such as the fact that while software simulations provide valuable insights into loop behavior, the actual fabrication of loops in clinical settings might not align perfectly with these predictions. Consequently, outcomes derived from analytical methods might exhibit discrepancies when translated into clinical scenarios. The frictional forces generated within the wire-to-bracket interface play a significant role in orthodontic mechanics. Regrettably, this interface was not incorporated into the study's simulation, potentially leading to an incomplete representation of the real-world frictional force system. The study omitted the influence of critical clinical factors, such as masticatory forces and other real-world conditions. These factors have the potential to impact the overall behavior of the orthodontic system but were not factored into the analysis, potentially limiting the study's practical applicability. These limitations underscore the need for comprehensive investigations that not only incorporate software simulations but also consider the complexities of actual clinical scenarios. A more holistic approach, encompassing fabrication variability, wire-bracket interactions, and clinical conditions, would yield a more accurate and insightful understanding of orthodontic mechanics. 

## Conclusions

After careful analysis, we have concluded that the MV loop demonstrates exceptional efficiency and an ideal M/F ratio for facilitating the translational movement of teeth. Its performance surpassed that of the Opus loop, both of which exhibited insufficient M/F ratios in the absence of pre-activation bends. The MV loop, with its higher M/F ratio in comparison to the other loops, proves to be a valuable tool for achieving effective translational movement of teeth during orthodontic treatment. To ensure the proper utilization of the MV Loop, we recommend its preparation using a 0.019 x 0.025-inch TMA wire. This selection will maximize the loop's capabilities and enhance its performance in achieving the desired tooth movement.
